# On How Psychophysical Thresholds are Altered by Unilateral Brain Injury Due to Stroke

**Published:** 2021-07-31

**Authors:** Melissa Allen, Tracy Kretzmer, George Jewell, Heather Murphy, Jeff Thostenson, Mark Mennemeier

**Affiliations:** 1 Department of Physical Therapy, University of Central Arkansas, USA; 2 Haley Veterans Hospital, USA; 3 Department of Neurology and Rehabilitation Medicine, University of Cincinnati, USA; 4 Clinical Solutions Group (CSG, Inc.), USA; 5 Department of Biostatistics, University of Arkansas for Medical Sciences, USA; 6 Department of Neurobiology and Developmental Sciences, University of Arkansas for Medical Sciences, USA

**Keywords:** psychophysical thresholds, absolute threshold, just noticeable difference, sensory impairment, neglect, lesion volume, functional independence, stroke, brain injury

## Abstract

**Objective:**

This study examined whether and how the absolute thresholds and the just noticeable difference thresholds for eleven, sensory/perceptual continua are altered by unilateral left and right hemisphere lesions due to stroke relative to healthy subjects.

**Methods:**

The three subject groups were those with unilateral right hemisphere lesions (n=21), with unilateral left hemisphere lesions (n=13), and age-matched control subjects (n=76). Absolute thresholds of sensory detection and just noticeable difference thresholds were assessed for perceptual continua spanning the visual, tactile, proprioceptive, thermal, and gustatory sensory modalities. For stroke subjects, brain lesions were analyzed using subtraction techniques and volume analysis with the MRIcro and MRIcroN software programs. Stroke subjects also complete tests for spatial neglect, stroke severity and functional independence.

**Results:**

There was no significant difference among subject groups regarding gender, race, hand dominance, age, or educational composition. There was no significant difference between subjects with right and left hemisphere lesions on measures of function, stroke severity, or lesion volume except for those with spatial neglect. The RHL group had a higher percentage of impaired perceptual continua (16%) than both normal controls (4%) and the LHL group (9%). If a stoke subject had an impaired threshold on one side of the body, they were ~5 times more likely to have an impaired threshold on the other side of the body. This result was more consistent and even exaggerated (~8 times more likely) in the small percentage of normal control subjects who demonstrated “impaired” sensory thresholds. Lesion volume was positively correlated with stroke severity and sensory threshold impairment, and it was negatively correlated with functional independence.

**Conclusions:**

When subjects, have difficulty detecting and discriminating sensory experiences, they tend to do so on both sides of the body. Unilateral right hemisphere stroke appeared to increase the relative frequency of altered thresholds occurring on the contralesional side of the body even though they made errors on both sides.

## Introduction

Psychophysics and neuropsychology are two classical approaches for examining and understanding sensory perception. Psychophysics focuses on the development of methods for quantifying the input/output relationship between physical stimulation and sensory experience (e.g., the absolute threshold (AB) for stimulus detection and the just noticeable difference (JND) between two stimuli) [1]. Neuropsychology uses test paradigms to reveal how sensation may be altered by damage to the nervous system [2]. Rarely, however, are the two approaches combined. The “separation” of these approaches probably relates to their distinct goals. In attempts to quantify stimulus-response relationships, psychophysics has focused on perception in normal subjects, and it has largely adopted a “black box” approach toward the nervous system. Neuropsychology, in contrast, attempts to correlate abnormalities in sensation with lesion characteristics (e.g., laterality, location, and volume) in order to learn how the nervous system mediates sensory perception.

The present manuscript represents a combined approach. We examined whether and how the AB and JND thresholds for eleven, sensory/perceptual continua are altered by unilateral left and right hemisphere lesions due to stroke. This study was a retrospective analysis of a dataset collected for a large, prospective study of magnitude estimation in patients with unilateral stroke who either did or did not have spatial neglect. The magnitude estimation data have been summarized elsewhere [3]. The AB and JND threshold data used in this study were collected as part of the larger investigation to ensure that the patient’s sensory abilities were sufficient to perform tests of magnitude estimation. Threshold data were also collected for a large sample of normal control subjects to establish the limits of normal performance.

These data are relevant to a long-standing question in neuropsychology concerning whether unilateral brain lesions can (or should) cause ipsilesional as well as contralesional sensory errors and whether one or both cerebral hemispheres contain representations of sensory events on both sides of the body. A now classical paper by Arthur Benton [4] addressed these questions, however, to our knowledge, they have not been resolved. Benton observed that while the clinical neurological exam is based on the doctrine of contralateral innervation, that sensory and motor function on one side of the body is mediated by the opposite cerebral hemisphere. Early clinical case reports and studies in the 1920s & 1930s occasionally reported patients with ostensibly unilateral left and right hemisphere lesions who exhibited bilateral sensory errors on the neurological exam, or errors on only the ipsilesional side of the body [5]. These findings raise concern that the clinical test method, particularly when applied in the acute stage of stroke, may be biased toward finding contralesional rather than ipsilesional sensory impairment because the method involves comparing sensation on two sides of the body and on using the ipsilesional side as a reference for “normal” perception. This procedure may bias the observer to focus on the contralesional side and ignore ipsilesional errors when present. Later studies beginning in the 1960s, which are summarized in Benton’s chapter [4], addressed some of these concerns by using standard methods in chronic stroke patients and by incorporating normal control subjects as a reference. Their results made it clear, however, that both ipsilesional and contralesional sensory deficits can be observed following unilateral brain lesions of either hemisphere, depending in part on the nature of the sensory task (e.g., two-point discrimination versus perception of movement direction). This result was surprising given the doctrine of contralateral innervation and claims were made for the cerebral dominance of somesthesis for both the left and right hemispheres. Presently, several explanations for ipsilateral and contralateral sensory errors after unilateral lesions seem plausible: 1) each hemisphere may have sensory representations for both sides of the body, 2) one hemisphere might be dominant for a given type of sensory function and damage to that hemisphere may result in bilateral sensory errors on some but not all types of sensory tests, and 3) lesion variables such as stroke chronicity, location (e.g., subcortical versus cortical) and volume, or the presence of comorbid behavioral syndromes like spatial neglect, could influence the laterality of sensory errors. These factors have not been examined systematically.

The present study had three aims: 1) to determine the prevalence and the laterality of altered sensory thresholds in patients with unilateral left and right hemisphere lesions due to stroke relative to the performances of normal control subjects, 2) to determine whether and how specific lesion characteristics relate to altered sensory thresholds, and 3) to determine how altered sensory thresholds relate to spatial neglect, functional status, and stroke severity. To test these aims, AB and JND thresholds were analyzed for patients with unilateral right hemisphere stroke, patients with unilateral left hemisphere stroke, and normal control subjects. Patients with stroke also completed standardized tests of functional independence, stroke severity, and of spatial neglect. Finally, the CT and MRI scans for 13 patients with right hemisphere stroke were analyzed to determine how lesion laterality, location and volume influenced the sensory thresholds. To anticipate our results, when subjects (even normal subjects) have difficulty detecting and discriminating sensory experiences, they do so on both sides of the body. Unilateral right hemisphere stroke appeared to increase the relative frequency of altered thresholds occurring on the contralesional side of the body.

## Materials and Methods

### Participants

The three subject groups were those with unilateral right hemisphere lesions (n=21), with unilateral left hemisphere lesions (n=13), and age-matched control subjects (n=76). Participants ranged in age from 33 to 86 years (mean age 58.6 years). All subjects with stroke were at least one-month post lesion onset. Subjects who demonstrated clinical and behavioral evidence of a first time or recurrent stroke were eligible for participation in this study. General assessment of IQ (Weschler Adult Intelligence Scale), memory (Weschler Memory Scale-Revised), and language (Western Aphasia Battery) were performed prior to testing to provide baseline data. The study excluded subjects who could not respond appropriately to commands due to aphasia or cognitive deficits. Subjects completed the Behavioral Inattention Test (BIT) to classify them as having or not having spatial neglect (i.e., a composite BIT score below 130 and clear evidence of contralateral omissions on at least one subtest) [6]. They completed the NIH Stroke Scale to measure stroke severity and the Barthel Index to measure functional independence in terms of activities of daily living [7,8]. Of the total population of participants with stroke, twenty-seven subjects (n=18, right hemisphere lesion; n=9 left hemisphere lesion) had either CT or MRI scans that could be used in lesion subtraction analysis.

### Absolute threshold procedures

Absolute thresholds of sensory detection were assessed for eleven perceptual continua spanning the visual, tactile, proprioceptive, thermal, and gustatory sensory modalities (Table 1 lists specific continua assessed). Two trials of each stimulus were performed for all absolute threshold assessments: one ascending trial and one descending trial following standard procedures for the “method of limits” [1].

### Just noticeable difference procedures

The just noticeable difference (JND) threshold is a statistical measure that identifies the smallest detectable magnitude of difference between a standard and comparison stimulus. Two trials were performed for JND assessment of each continua: one in ascending order and one in descending order using the method of limits and taking the average of the two trials as the JND. The standard stimulus intensity that was selected for the JND trials of each continua was greater than the smallest value used for absolute threshold assessment to ensure subjects would be able to detect the standard stimuli for all continua (Table 2).

As with absolute threshold assessments, tactile, proprioceptive, and thermal continua were assessed bilaterally. The subject was given a rest period of at least 2 seconds between presentations of each stimulus, the length of rest between each presentation varied by continua. Due to time requirements for testing and the abilities of some subjects, not all subjects were able to be assessed on all continua.

### Statistical analysis

Thresholds that were elevated relative to those of normal control subjects were considered as impaired for this study. All normal control subjects performed absolute and just noticeable difference threshold assessments for all perceptual continua described above. Raw threshold scores for each continuum of subjects with stroke were converted to z-scores using the mean and standard deviation of normal control subjects. A *z*-score of 1.96 or greater was coded as an elevated (impaired) threshold, and a *z*-score of −1.96 or less was coded as a decreased (below normal) threshold. (Note: Due to the procedural calculations of the roughness threshold, a *z*-score of 1.96 or higher was coded as a decreased threshold while a *z*-score of −1.96 or higher was coded as an increased threshold). Once each subject’s scores were coded, the frequency and magnitude of impairment was calculated.

To allow for comparison across groups, *prevalence of sensory impairment* was defined as the percentage of impaired threshold assessments relative to the number of assessments completed by that subject. Prevalence of sensory impairment was examined as follows: 1) prevalence of impairment of absolute threshold, 2) prevalence of impairment of just noticeable difference, 3) prevalence of impairment in each of the eleven perceptual continua, and 4) prevalence of sensory impairment in normal control subjects, subjects with RHL, and subjects with LHL. Potential differences in prevalence of sensory impairment of each of the eleven perceptual continua in subjects with brain lesions were investigated using a chi square analysis with a 2 × 3 contingency table of lesion side (LHL vs. RHL) vs. continua outcome [below the 95% cut off (impaired), within normal limits, and above the 95% cut off (normal)].

A generalized linear model was used to determine if there was a difference in prevalence of sensory impairment between subjects with right hemisphere lesions, subjects with left hemisphere lesions, and normal control subjects. A one-way ANOVA was used with group assignment (LHL, RHL, normal control) as the predictive factor for response variables for percentage of impaired thresholds (i.e., percent failures=number failed/number completed × 100).

A generalized linear model was used to determine how lesion laterality influenced the magnitude (or severity) of sensory impairment. The mean and median *z*-scores for each group were analyzed. While the mean provided the average of *z*-scores for each group, given the small sample size in this study the median z-scores were less sensitive to potential outliers.

Odds ratios were used to understand whether and how right and left hemisphere lesions predict unilateral or bilateral sensory impairment. Logistical regression models were run firstly to predict contralesional impairment and secondly to predict ipsilesional impairment for the overall sample of subjects and for each subject group.

### Lesion analysis

Brain lesions were analyzed using subtraction techniques and volume analysis with the MRIcro and MRIcroN software programs. Clinically obtained CT or MRI images were transferred to a standard Montreal Neurological Institute (MNI) template image while adjusting for the angle of cut of the original image. For subjects whose lesions were transferred either from film or from digital images of horizontal slices, the clinically obtained CT or MRI image and the ch2better.nii MRIcroN template were opened in MRIcro. The MNI template image was rotated to match the yaw, pitch, and roll values of the original image. For those subjects whose CT or MRI data were obtained from DICOM files, the DICOM files were loaded in MRIcroN. The clinically obtained image was rotated to match the yaw, pitch, and roll of the MNI template. The lesions were mapped from the clinical scan to the template images. Regardless of method, regions of interest (ROI) were created on template images by physically drawing brain lesions from each slice of the clinical scan onto the corresponding template images on MRIcroN using anatomical landmarks as guides. A 3-D model of the lesion was created by smoothing ROIs using FWHM value of 8 and a threshold of 0.05.

Patients were classified for the subtraction analysis as falling into either a low impairment group (impairment in less than 12% of threshold assessments, n=6) or a high impairment group (impairment in more than 12% of threshold assessments, n=7). This classification provided a necessary separation between subjects in terms of the frequency and severity of their sensory impairment.

## Results

There was no significant difference among subjects regarding gender, race, hand dominance, age, or educational composition. There was no significant difference in subjects with right and left hemisphere lesions on measures of function, stroke severity, or lesion volume (Table 3).

Subjects who had neglect had greater stroke severity, lower functional status, and larger lesion volumes than subjects who did not have neglect (Table 4).

### Lesion laterality and prevalence of sensory impairment

**Figure 1.**
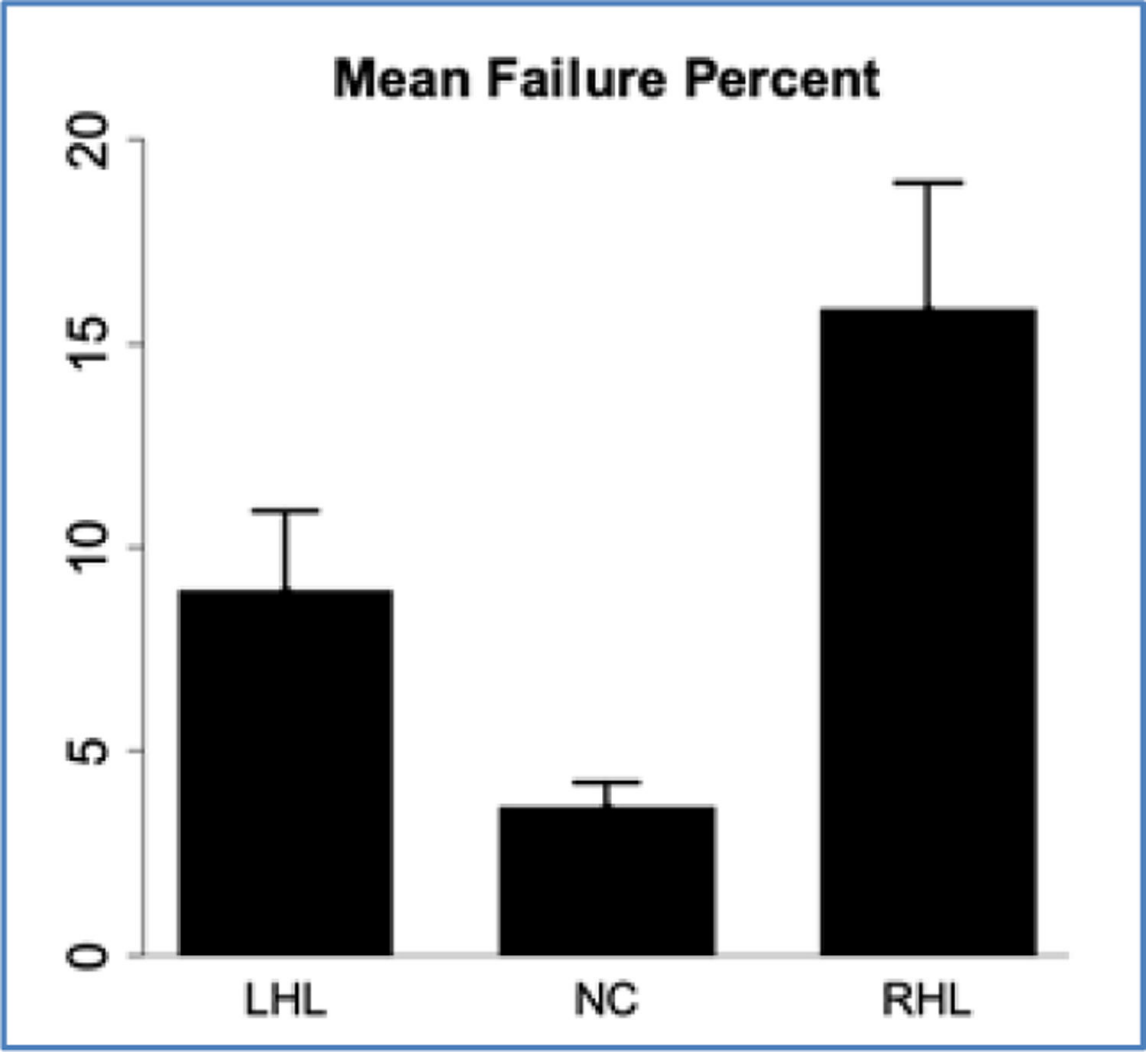
Mean Failure percentage by group

Overall results of the ANOVA model comparing the percent of impaired continua revealed a significant effect of group (df=2, F=19.81), *p*<0.001. The Tukey-Kramer adjustment was applied to correct for multiple comparisons. The RHL group had a higher percentage of impaired continua (16%) than both normal controls (4%) *p*<0.0001 and the LHL group (9%) *p*=0.04. Normal controls and subjects with LHL did not differ from each other (p=0.07). Figure 1 displays the mean percentage of impairments for each group.

Therefore, subjects who have sustained a right hemisphere lesion have a significantly greater prevalence of sensory threshold impairment than normal control subjects and subjects with left hemisphere lesions. Supplemental Figures 1–6 (included in supplemental materials) provide histograms to display the frequency of overall errors of absolute and just noticeable difference thresholds. These reveal that most subjects do not demonstrate impaired threshold tests, so it is important to identify factors associated with impairment of sensory thresholds.

**Figure 2.**
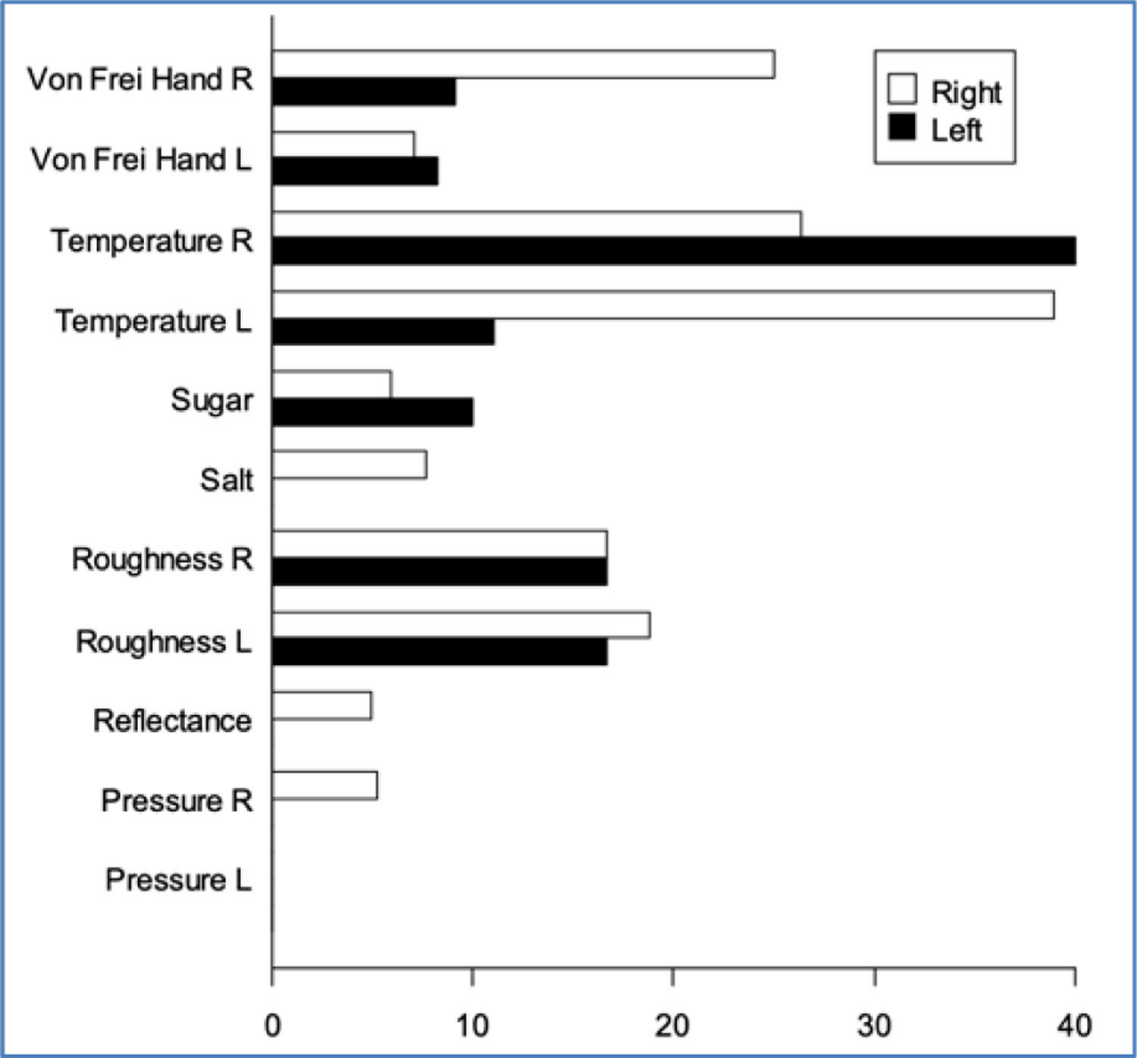
Percentage of impairment in just absolute threshold continua

**Figure 3.**
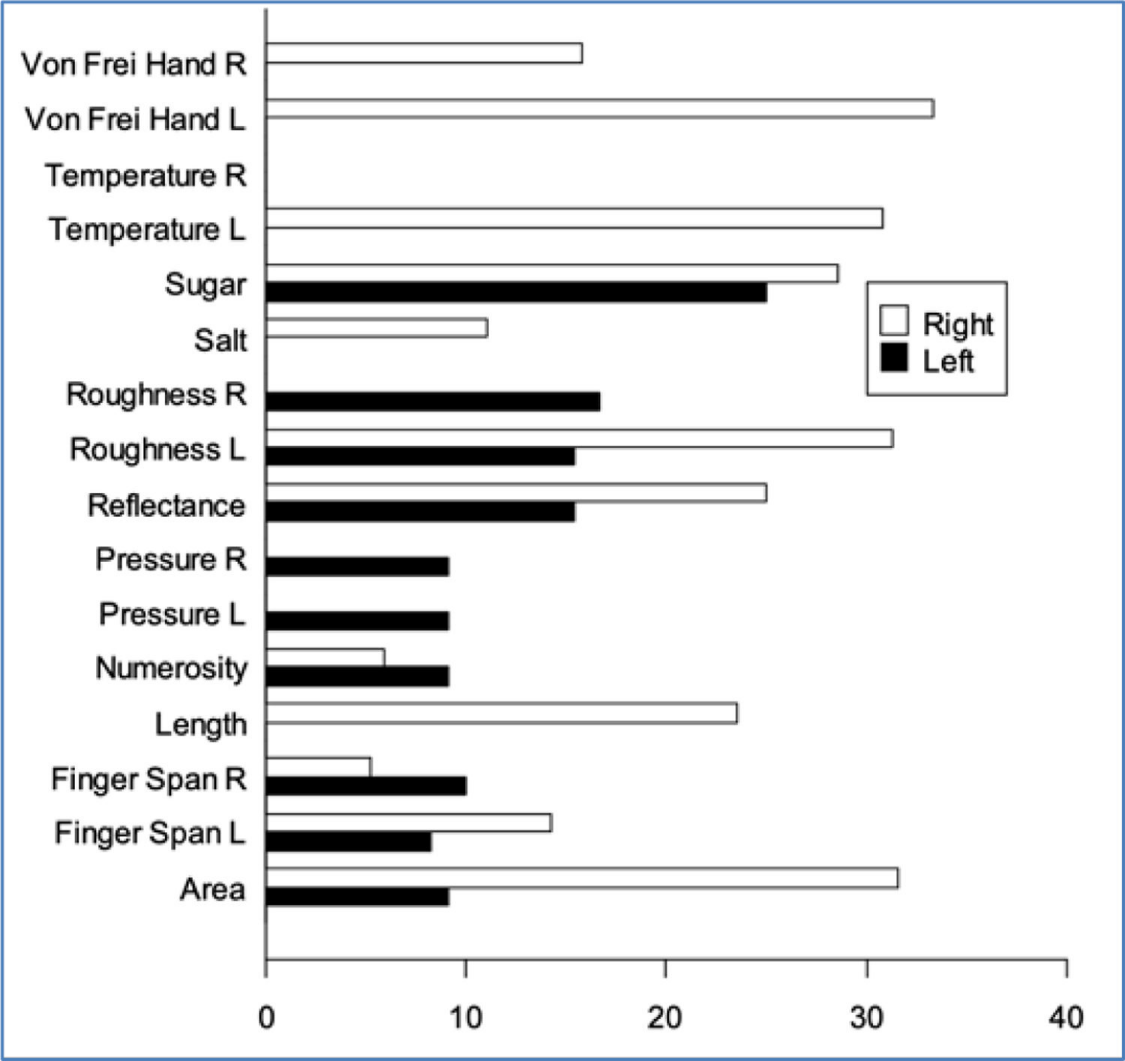
Percentage of impairment in just noticeable difference continua

To determine if there was a significant difference in prevalence of sensory impairment among the eleven perceptual continua, an overall chi-square analysis comparing subjects with right and left hemisphere lesions in terms of overall percent impairment was performed and found to be significant (df=2, X^2^=9.20, *p*=0.01). When the chi-square analysis was performed for each continua for absolute and just noticeable difference threshold assessments separately, a significant effect was found in impairment of just noticeable difference of von Freir hair assessment to the left hand in subjects with right hemisphere lesions, (df=1, X^2^=4.17, *p*=0.04) indicating this continua had a greater prevalence of impairment than other continua. Figures 2 and 3 display histograms showing the percent of impaired absolute and JND thresholds for the subjects with right and left hemisphere lesions in each perceptual continua. Overall, just noticeable difference assessments are impaired more often than absolute threshold assessments

**Figure 4.**
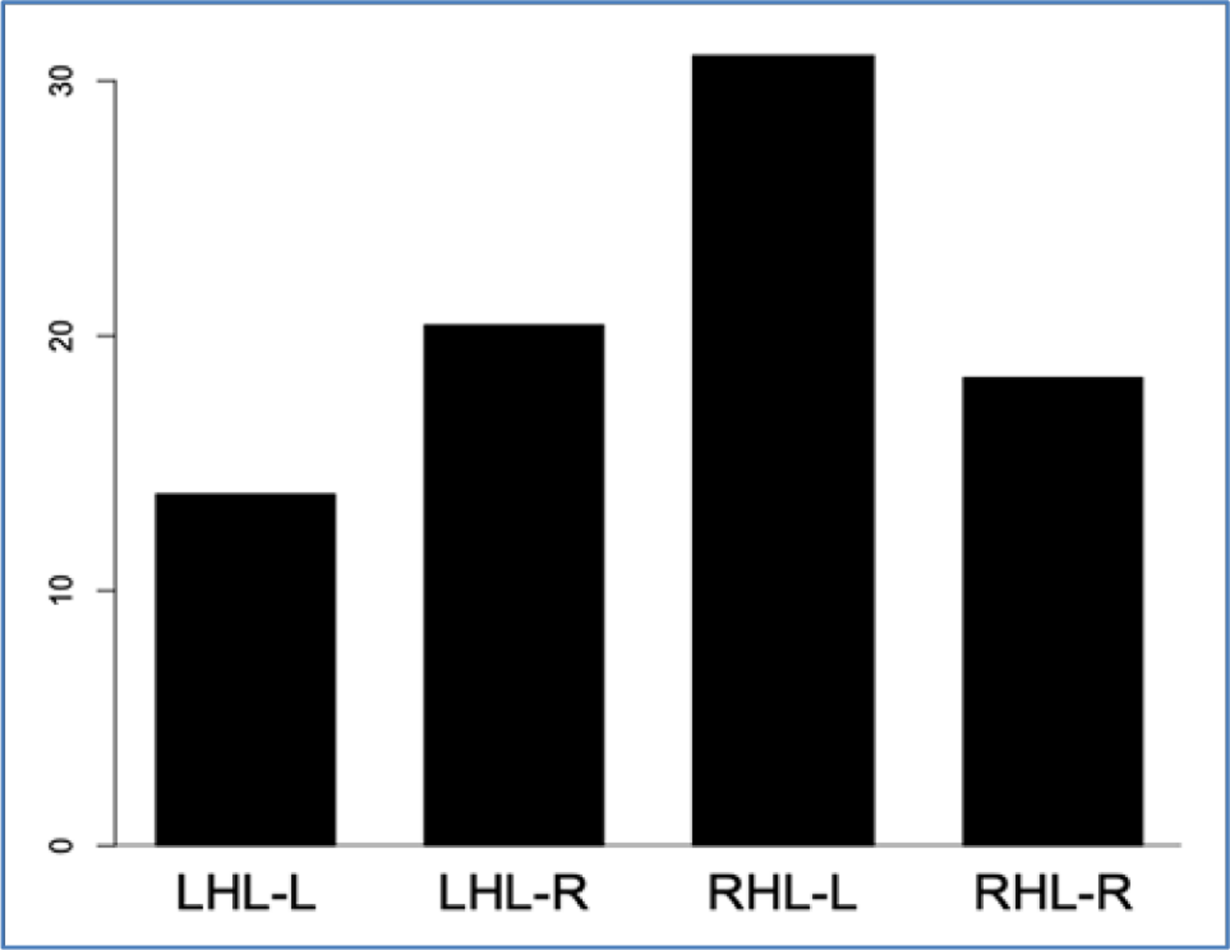
Percentage of failures by lesion laterality and side of error

To further examine how lesion laterality accounted for errors occurring on one or the other side of the body, log transformations of each subjects’ raw scores were calculated to obtain normal residuals. Overall, the RHL group demonstrated higher log-percentage of impaired thresholds than the LHL group (df=25, t=-1.98, p=0.0587). When examining the interaction of group and side of error, the RHL group made more left sided errors than did the LHL group (df=9, t=-3.44, *p*<0.03, Tukey-Kramer), indicating subjects with right hemisphere lesions have impairment of more continua on the left side of the body than subjects with left hemisphere lesions. Transforming this data back to units of percentage of impaired thresholds, it can be seen that on average subjects with right hemisphere lesions demonstrated impairment of left sided continua 2.26 times more often than subjects with left hemisphere lesions demonstrated impairment of left side continua. Figure 4 provides a histogram to show the tendency of subjects with right hemisphere lesions to have impaired thresholds of continua on the left side of the body more often than subjects with left hemisphere lesions.

### Lesion laterality and severity of sensory impairment

**Figure 5.**
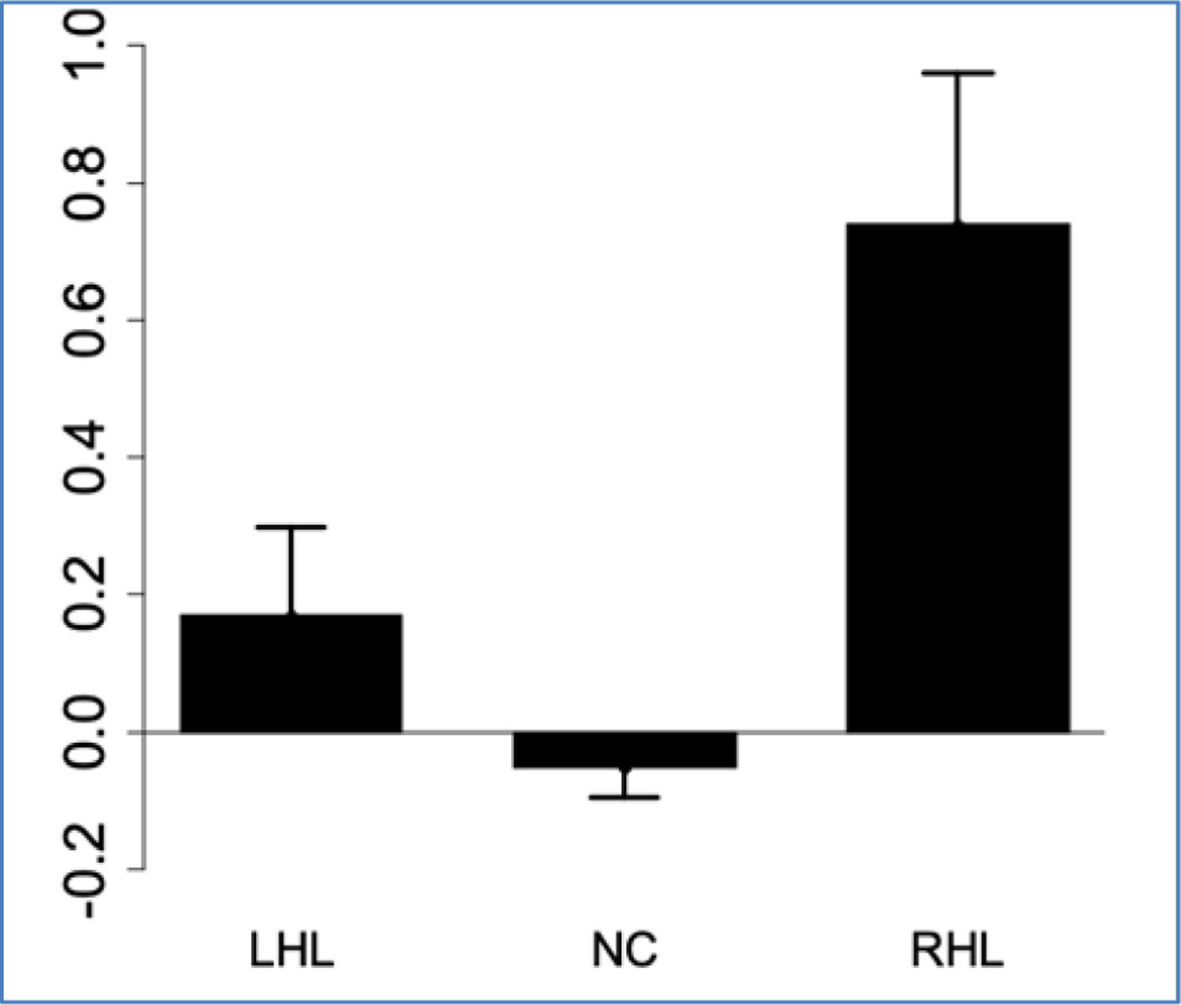
Mean *z*-scores of each group

ANOVA models compared severity of sensory impairment of the study groups in terms of both the mean and median z-scores. For the mean *z*-score, there was a significant effect of group (df=2, F=19.33, *p*<0.0001). Using a Tukey-Kramer adjustment, the right hemisphere lesion group had a higher mean *z*-score value (0.76) than the normal control subjects (-0.02, *p*<0.0001) and the subjects with left hemisphere lesions (0.14) *p*<0.003. Figure 5 displays the mean z-scores of each group.

A similar pattern was observed for the median *z*-score. There was a significant effect for group (df=2, F=12.18, *p*<0.0001). The Tukey-Kramer adjustment revealed that subjects with right hemisphere lesions had a higher median *z*-score (0.31) than normal control subjects (-0.12) *p*<0.0001 and subjects with left hemisphere lesions (-0.03), *p*<0.01. Normal control subjects and subjects with left hemisphere lesions did not differ from one another in terms of median or mean *z*-score.

### Prediction of bilateral sensory impairment

Odds ratios were calculated using logistical regression to determine if an impaired threshold on one side of the body predicted an impaired threshold on the opposite side of the body. When normal control subjects and subjects with stroke were considered together, the odds ratio estimate was 4.69 and the 95% confidence limit excluded 1 (i.e., spanning 1.99, 11.05). This finding suggests that if a subject had an impaired threshold on one side of the body, they were 4.69 times more likely to have an impaired threshold on the other side of the body. This finding was more consistent in normal control subjects whose odds ratio was 7.94 (confidence limits=2.39, 26.31). When calculated for the RHL and LHL subjects together or alone, the 95% confidence intervals included 1 and thus were not significant (Table 5).

A chi-square analysis was performed to determine if impairment of continua on one side of the body made a subject more likely to demonstrate impairment of continua on the other side of the body. There was a significant effect seen for normal subjects (df=1, X^2^=13, *p*=0.0003) but not for subjects with either a left or right hemisphere lesion.

These results suggest that when subjects (even without brain lesion) have difficulty detecting and discriminating sensory experiences, they do so on both sides of the body. Unilateral right hemisphere stroke appeared to increase the relative frequency of impaired thresholds occurring on the contralesional side of the body.

### Impact of unilateral neglect and sensory impairment on functional performance

In subjects with right hemisphere lesions, a significant correlation was found between the collective mobility subscores of the Barthel Index (mobility, transfer, and stairs) and the median z-score on absolute threshold assessments n=17, r=-0.52, *p*=0.0325. A significant correlation was also found between the absolute median *z*-score and transfer score from the Barthel Index n=17, r=-0.55, *p*=0.02. When subjects with right and left hemisphere lesions are examined together, there is a significant positive correlation between the percentage of impairment on absolute threshold assessments and the total score on the NIH stroke scale n=28, r=0.39, *p*=0.039. The correlation between overall percentage of impaired continua on all threshold assessments and the NIH stroke scale score had a *p*=0.07 (n=28, r=0.35). The subscale score assessing extinction inattention on the NIH stroke scale correlates with the percentage of impairment on absolute threshold assessments with n=25, r=0.37, *p*=0.07.

Correlations were also performed between lesion volume and the identified functional assessments. When subjects with right and left hemisphere lesions were analyzed together, lesion volume and the NIH stroke scale had a significant positive correlation, n=17, r=0.49, *p*=0.04. The association between the Barthel Index and lesion volume is n=17, r =-0.46, with *p*=0.06. Lesion volume is correlated with functional status and stroke severity as determined by the NIH stroke scale; and stroke severity also correlated with the percentage of impaired sensory threshold assessments (Table 6 and 7). As lesion volume increases, stroke severity also increases while functional independence decreases. As stroke severity increases, the percentage of impairment on absolute threshold assessments also increases.

### Lesion analysis

**Figure 6.**
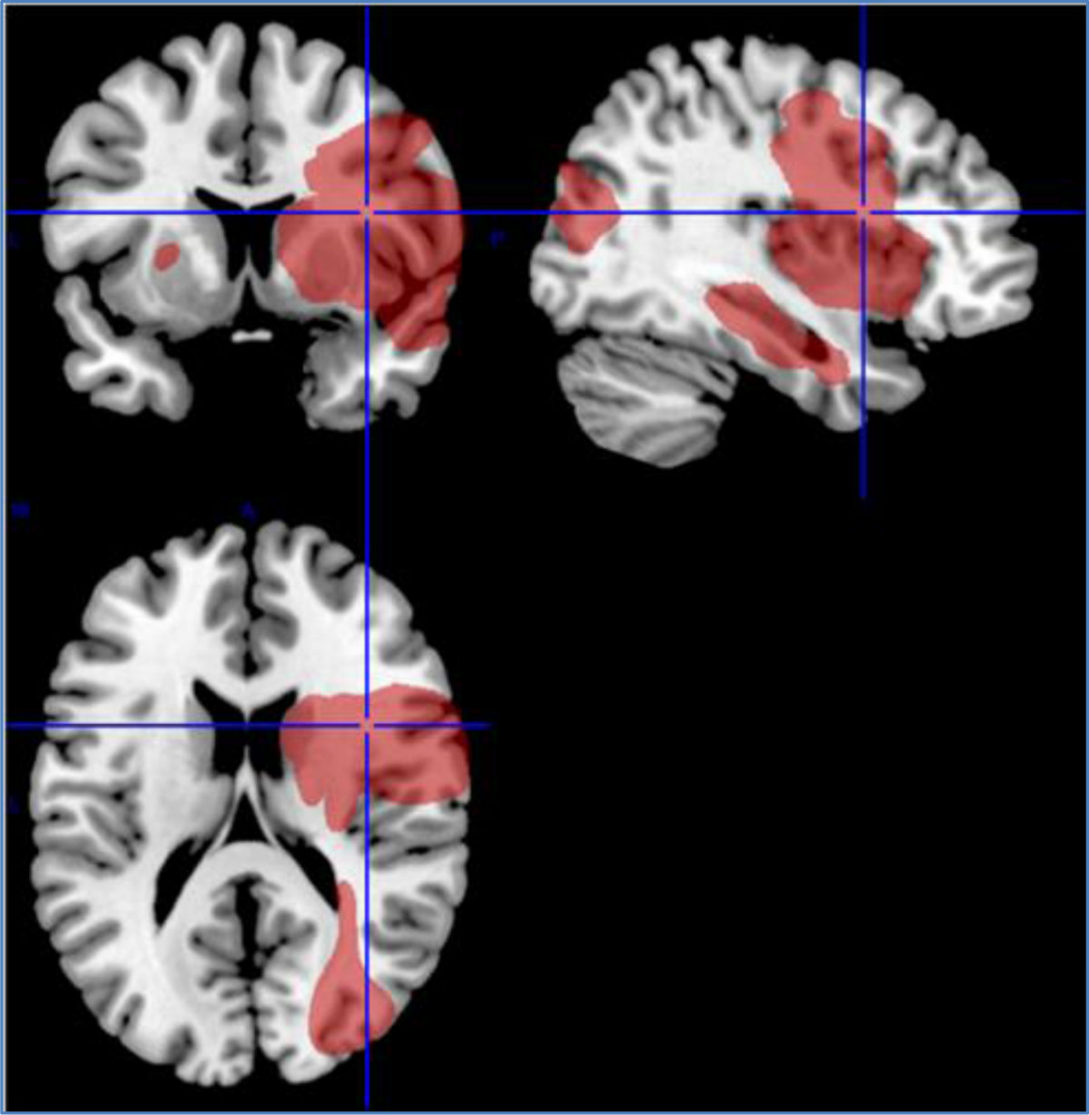
Lesion overlay images from MRIcron software

**Figure 7.**
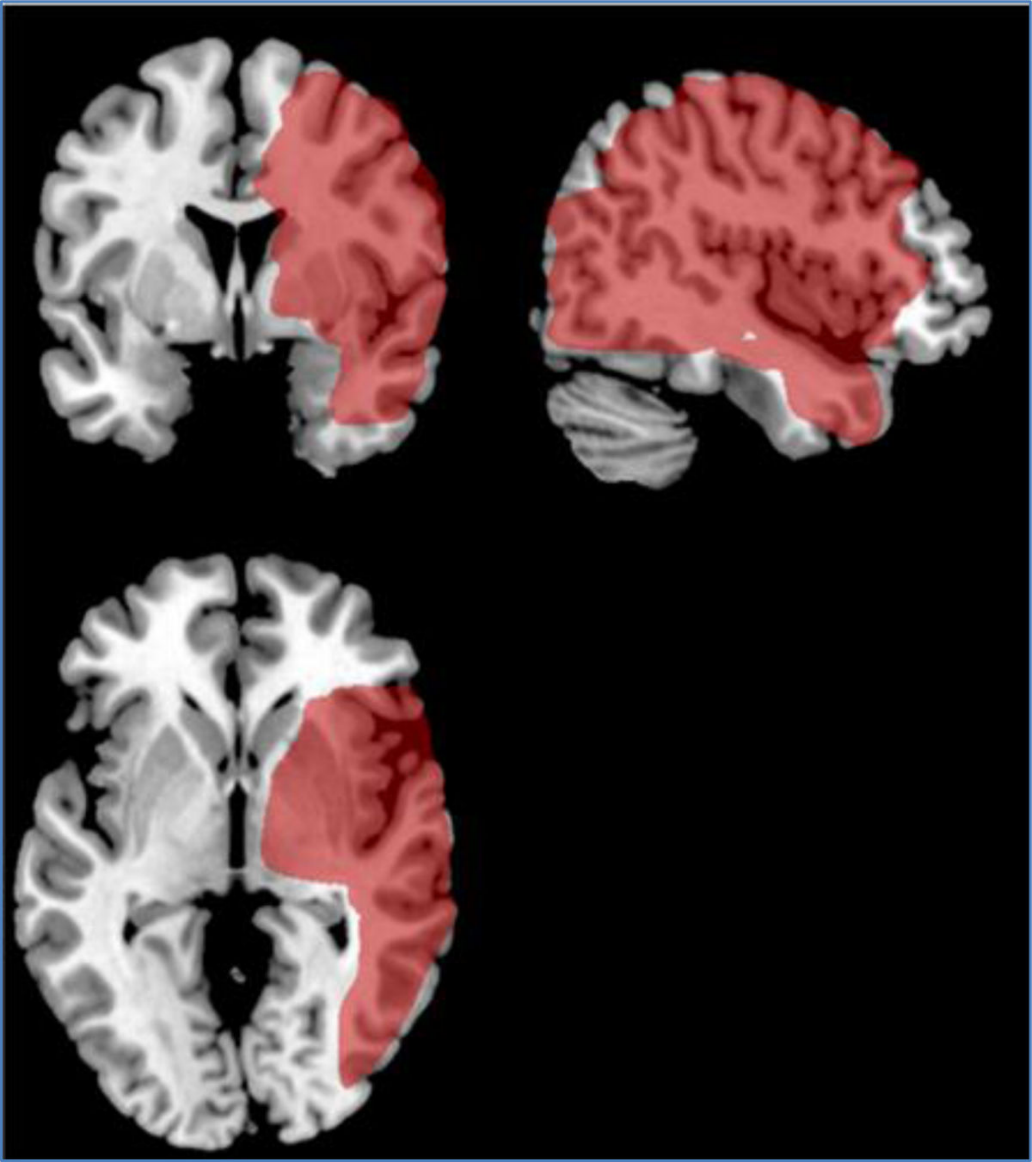
Lesion overlay images from MRIcron software

Lesion subtraction analysis was performed to examine the effect of lesion volume on the percent of impaired continua. Only patients with right hemisphere lesions were considered because there were too few patients with scans in the left hemisphere lesion group to perform a meaningful lesion subtraction analysis. In fact, the subtraction analysis for the subjects with right hemisphere lesions can only be considered preliminary due to the small sample size. Subjects with right hemisphere lesions (n=13) were split into two groups – those who had 12% or more impaired continua (high impairment group, n=7) versus those who had less than 12% impaired continua (low impairment group, n=6). There was no significant difference in lesion volume between the high (21cm^2^) and low (102cm^2^) impairment groups (mean difference=80.67 (SD=69.78), *p*<0.11, *t*-test for unequal variance) even though lesion volume was five times larger in the low impairment group. Figures 6 and 7 display the lesion overlays obtained using the MRIcroN program.

**Figure 8.**
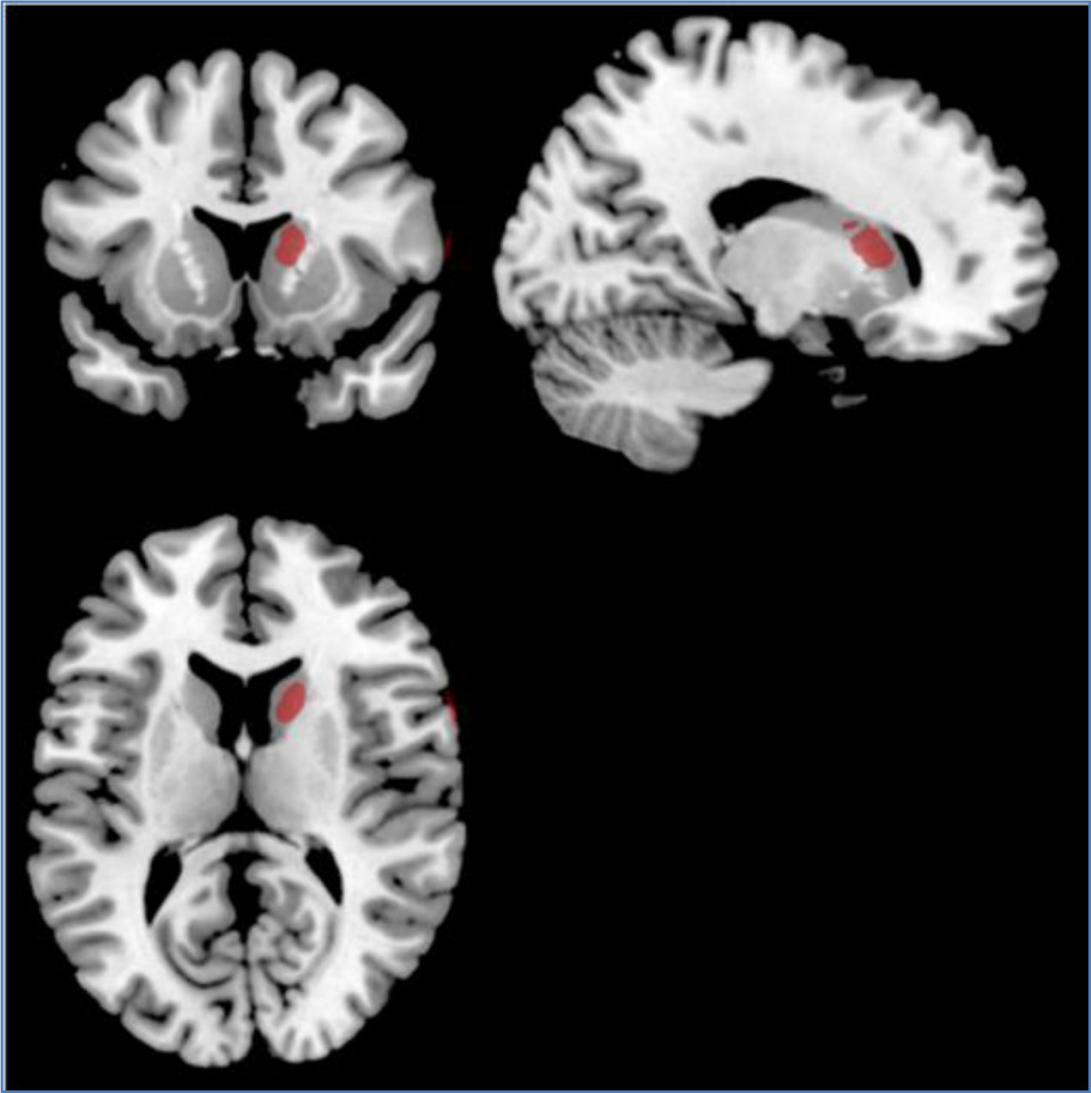
Lesion subtraction images

Subtraction analysis identified the caudate nucleus as the lesion site that was most common to at least 29% of patients with right hemisphere lesion in the high impairment group (>12% impaired continua) and not common to patients with right hemisphere lesion in the low impairment group (<12% impaired cotinua). See Figure 8 for subtraction images.

## Discussion

Regarding the presence of altered sensory thresholds in patients with left and right hemisphere lesions, we found that altered sensory thresholds were observed following stroke of stroke regardless of lesion laterality, but particularly among subjects with right hemisphere injury. Altered sensory thresholds were defined as those which exceeded a *z*-score of +/− 1.96 based on the mean and SD of normal control subjects. As expected, based on this criterion, about 4% of normal control subjects had thresholds exceeding a *z*-score of +/−1.96. In contrast, 16% of subjects with right hemisphere lesions and 9% of subjects with left hemisphere lesions exceeded this value, representing elevated sensory thresholds. Also, whereas the percent of impaired thresholds was significantly greater for subjects with right hemisphere lesions than for normal control subjects; subjects with left hemisphere lesions were not significantly different from normal control subjects even though their percent of impaired thresholds was twice as high.

Regarding whether and how sensory thresholds are altered in association with volume and location of right hemisphere lesion, there was no significant difference in lesion volume between subjects with high (>12%) and low (<12%) rates of impairment on sensory threshold assessments. If anything, impairment of sensory thresholds was associated with smaller lesions involving the caudate nucleus. Subtraction analysis revealed that a lesion in the caudate nucleus was common to at least 29% of the subjects in the high impairment group and not common to subjects in the low impairment group (but appropriate cautions apply as mentioned further below).

Regarding whether altered sensory thresholds are related to functional status, a greater percentage of impaired absolute thresholds correlated with an increase in the extinction subscale of the NIHSS indicating greater severity of deficit. Additionally, the overall severity of sensory impairment as measured by the median *z*-score correlated with a decrease in functional independence on mobility tasks (i.e., ambulation, transfers, and ascending/descending stairs), in subjects with right hemisphere lesions. As expected, based on earlier studies, lesion volume correlated significantly with both stroke severity on the NIHSS and functional independence as measured by the Barthel Index total score. Unlike the association between impaired sensory thresholds and lesion volume, large lesion volume was correlated with greater functional impairment and greater stroke severity. So, sensory impairment is related to decreased functional ability, but this relationship is not simply due to having large lesions. Rather, a critical lesion location may be more important.

Certain methodological limitations may limit the generalizability of our findings. The procedural assessments of absolute and just noticeable difference thresholds were limited to the tests used in a larger study of magnitude estimation. They did not include more traditional neurological assessments like vibration sense and may not generalize well to a neurological exam. Additionally, subjects were actually required to have sensory function sufficient to perform a test of magnitude estimation. As a neurological exam is performed, in part, to detect primary deficits in sensory function, such subjects would have been excluded for a given sensory domain in this study. Nor did the sensory evaluations include the lower extremity. A limitation regarding lesion analysis is that only thirteen subjects could be analyzed. A more thorough approach for subtraction analysis requires comparison of about 20 subjects or more. We did not perform a lesion analysis of patients with left hemisphere lesions because too few scans were available. Additionally, even if more scans were available for left hemisphere subjects, large left hemisphere lesions would preclude the assessment of sensory thresholds in patients with aphasia because they would not be able to communicate their responses. So, a true comparison of patients with right and left hemisphere patients using subtraction procedures would be hard to achieve.

Even with these limitations, however, this study is novel in several respects. First, no study to our knowledge has examined classical psychophysical thresholds in stroke patients or attempted to relate these deficits to functional ability. Nor have normal control subjects underwent all of the sensory threshold assessments and served as the basis for defining sensory impairment. This approach produced an interesting result. Whenever normal control subjects had elevated thresholds, they were almost five times more likely than subjects with stroke to make errors on both sides of the body. Contrary to the neurological exam, which uses the ipsilesional side of the body as a normal reference, we found that when sensory thresholds are impaired, they are more, rather than less likely, to be impaired on both sides of the body. Unilateral brain injury changes this relationship by elevating the frequency of altered thresholds observed on the contralesional side of the body. For example, subjects with right hemisphere lesions made errors on both sides of the body, but they had a greater number of contralesional errors than did subjects with left hemisphere lesions who also made errors on both sides of the body. Lesion laterality also impacted the severity of sensory impairment, as subjects with right hemisphere lesions were found to have higher mean and median *z*-scores on threshold assessments than normal controls and subjects with left hemisphere lesions. These findings present a new way of looking at sensory impairment following unilateral brain injury. Somewhat contrary to the doctrine of contralateral intervention, the findings of this study suggest that when sensory impairment is observed, it is likely to be observed on both sides of the body but perhaps more frequently and severely contralateral to brain injury.

This study also produced an unexpected result regarding the relationship, or lack thereof, between impaired sensory thresholds and unilateral neglect. Neglect is defined as an attentional deficit that influences sensory awareness for the contralesional side of the body; so, the relationship between neglect and altered sensory thresholds is intuitive [9]. The fact that neglect and impaired sensory thresholds were not correlated in this study; therefore, is surprising. Several factors may account for the absence of this relationship. First, the BIT was used to define neglect. The BIT relies heavily on visual stimuli. Had neglect been defined with greater emphasis on sensory tests, like extinction, the correlations between neglect and impaired sensory thresholds may have been stronger. In fact, the extinction scale on the NIHSS was significantly correlated with the percentage of impaired sensory thresholds. So, using the BIT as the sole definition of neglect may not have been the optimal means of testing whether impaired absolute and JND thresholds are related to neglect.

Secondly, all of the sensory testing was performed unilaterally in this study. Extinction in neglect is only observed upon double, simultaneous stimulation of both sides of the body. Extinction is also defined, in part, by a subject’s ability to perform unilateral stimulation at a level that approximates normal. So, the procedures used to test sensory thresholds in this study may not be sensitive to the influence of neglect on sensory processing. Had sensory testing been performed simultaneously, and bilaterally, then a greater correlation with neglect would be expected. Regardless, it is safe to conclude that sensory thresholds were not impaired in this study simply due to the presence of unilateral neglect.

Third, neglect is related, in part, to a subject’s ability to develop mental representations of sensory events at a cortical level but this may not explain how subjects failed sensory thresholds [10]. Sensory thresholds may have been impaired in this study, not because subjects failed to accurately represent stimulation at a cortical level, but rather because their ability to judge these sensory representations was impaired. For instance, the lesion analysis data suggests that impaired sensory thresholds were not due to the volume of damage to the cortex; but rather, to its location – most common when lesions involved the head of the caudate nucleus. The structures comprising the basal ganglia (caudate, putamen, and globus pallidus) are known to play a role in voluntary movement and cognitive function. The head of the caudate nucleus shares anatomical connections with the dorsolateral prefrontal cortex, a structure critical for cognitive processing, such as learning associations, regulating attention, and classifying stimuli, as well as other executive functions [11,12]. A study by Seger and Cincotta [11] examined the role of the caudate nucleus in learning classification. For this study, subjects were read a scenario telling them they would have to predict the weather outcome based on a visual stimulus that was presented. The examiner would then show the subject an image of arbitrary lines that were coded as either “rain” or “sun”, and the subject had to identify in which category the image belonged. These authors referenced previous studies that utilized fMRI and PET imaging to determine if/how the caudate nucleus is activated during tasks involving stimulus classification and other arbitrary responses to stimuli. The results of the study by Seger and Cincotta, and of those they reviewed, indicate that the head of the caudate nucleus is most active during tasks that involve feedback processing in response to a stimulus [11].

The fundamental procedures of stimulus interpretation in the study by Seger and Cincotta [11] are similar to those used in this study to assess sensory thresholds. Both studies required subjects to make a judgment based on a stimulus presented and to provide feedback to the examiner. The subjects of the present study, and particularly those who had lesions of the caudate nucleus, demonstrated impaired threshold assessments more often than normal subjects to classify the stimulus as present or significantly different than a comparison stimulus. Our results present an intriguing new hypothesis that the sensory thresholds were impaired due to a cognitive, rather than a sensory processing error. This may be why impaired sensory thresholds were not correlated with lesion volume or neglect but rather to lesions involving the caudate nucleus. To test this hypothesis, one could examine sensory thresholds prospectively in patients who have primary lesions of the caudate nucleus.

In summary, these data are consistent with Benton’s observations of ipsilesional and contralesional sensory errors following lesions of the right and left hemisphere [4]. They are also consistent with the observation that contralesional errors may be more severe and frequent than ipsilesional errors in patients who have unilateral brain injury, particularly in those with right hemisphere injury. They do not suggest, however, that both hemispheres necessarily have representations of both sides of the body, and this is why unilateral brain injury can cause ipsilesional as well as contralesional sensory errors. Approximately, five percent of our normal control subjects demonstrated impaired sensory thresholds by a statistical definition and when they had altered thresholds on one side of the body, they were five times more likely than stroke patients to have altered thresholds on the other side of the body. It is unlikely that they have a representational deficit as may be present in a stroke patient [10], rather, they probably just fail to discriminate well between different levels of sensory stimulation. Their fidelity of judgement may be lacking, and the same problem could alter sensory thresholds and sensory testing in stroke patients.

## Supplementary Material

FNNR-2-100014 Supplementary file

## Figures and Tables

**Table 1.** Absolute threshold procedures

Sensory Domain	Perceptual Continuum	Stimulus Description	Subjects Tested (n)
**Visual**	Line Length	Detection of a line: 7–21cm lines utilized	Control=39 LHL=10 RHL=16
	Area	Detection of box: Boxes with areas of 2.5cm^2^–6.5cm^2^ utilized	Control=39 LHL=10 RHL=16
	Numerosity	Detection of dots: 9–49 dots utilized	Control=39 LHL=10 RHL=16
	Reflectance	Detection of color on black poster board – various shades of white paint samples were used	Control=39 LHL=11 RHL=17
**Tactile**	Roughness (L/R)	Detection of texture of sandpaper grit. 100% cotton vellum and sandpaper with grits of 300, 500, 700, 900, 1100, 1180, 1260, 1280 were used.	Control=39/39 LHL=10/10 RHL =15/17
	Von Freir (L/R)	Detection of pressure from Von Freir hair. Von Freir sizes 0, 0.008, 0.02, 0.04, 0.07, 0.16, 0.4, 0.6,1.0, 1.4, 2.0 were used	Control=39/39 LHL=10/10 RHL=11/17
	Pressure	Detection of pressure from cuff on upper arm. Pressures of 0, <16, 16, 18, 20 used.	Control=39/39 LHL=11/11 RHL =13/18
**Proprioceptive**	Finger Span (L/R)	Detection of a block between the thumb and index finger.	Control=39 LHL=10 RHL=11
**Thermal**	Temperature (L/R)	Detection of temperature that differs from skin temperature	Control=39/39 LHL=8/9 RHL=15/16
**Gustatory**	Sugar	Detection of the presence of sugar in a solution. Solutions of the following molarities were used: 0.0, 0.37, 0.075, 0.15, 0.30, 0.45	Control=38 LHL=9 RHL=13
	Salt	Detection of the presence of salt in a solution. Solutions of the following molarities were used: 0.0, 0.2, 0.3, 0.4, 0.5	Control=39 LHL=9 RHL=15

**Table 2.** Just noticeable difference procedures

Sensory Domain	Perceptual Continuum	Stimulus Description	Subjects Tested (n)
**Visual**	Line Length	Differentiation of line length: A standard line length of 14cm was compared to lines ranging from 7–21cm.	Control=39 LHL=16 RHL=10
	Area	Differentiation of box area: A standard box of 4.5cm^2^ was compared to boxes ranging in area from 2.5–6.5cm^2^ (in 0.1cm^2^ intervals)	Control=39 LHL=10 RHL=16
	Numerosity	Differentiation of number of dots: A standard slide of 29 dots was compared to a slide with 9–49 dots	Control=39 LHL=10 RHL=16
	Reflectance	Differentiation of object reflectance. Reflectance values of 73.4%, 68.4%, 63.6%, 59.1%, 54.8%, 50.7%, 46.8%, 43.1%, 39.5%, 36.2%, 30.0%, 27.2%, 24.6%, 22.1%, 19.8%, 17.6%, 15.6%, 13.7% 12% were used	Control=39 LHL=11 RHL=17
**Tactile**	Roughness (L/R)	Differentiation of textures: A standard sandpaper grit of 1350 was compared to 100% cotton vellum and sandpaper of the following grits: 900, 1100, 1180, 1260, 1280, 1320, 1380, 1400, 1420, 1440.	Control=39/39 LHL=11/10 RHL =15/18
	Von Freir (L/R)	Differentiation of pressure from Von Freir hairs: A standard Von Freir hair (size 4.0) was compared to Von Freir hairs of the following sizes: 0.4, 0.6, 1.0, 1.4, 2.0, 6.0, 8.0, 10.0, 15.0, 26.0, 60.0	Control=39/39 LHL=8/8 RHL=12/16
	Pressure	Differentiation of pressures from cuff: A standard pressure of 80 was compared to pressures of 82, 84, 86, 88, 90, 92, 94, 96, 98, 100	Control=36/37 LHL=10/10 RHL =14/17
**Proprioceptive**	Finger Span (L/R)	Differentiation of finger span: A standard block with height of 1.9cm was compared to blocks of the following heights: 0.9, 1.0, 1.2, 1.4, 1.5, 1.75, 2.0, 2.0, 2.35, 2.5, 2.6, 2.7 (all cm)	Control=39/39 LHL=10/9 RHL=n/16
**Thermal**	Temperature (L/R)	Differentiation of temperature: Temperature apparatus on forearm with a standard temperature of 40°C was compared to temperatures ranging from 36–44°C	Control=37/38 LHL=10/9 RHL=11/16
**Gustatory**	Sugar	Differentiation of sugar concentration in a solution: A standard solution of 0.75M is compared to solutions with the following molarities: 0.60, 0.65, 0.70, 0.80, 0.85, or 0.90	Control=37 LHL=7 RHL=12
	Salt	Differentiation of salt concentration in a solution: A standard solutions of 0.27M is compared to solution with the following molarities: 0.21, 0.23, 0.25, 0.29, 0.31, 0.33	Control=37 LHL=6 RHL=9

**Table 3.** Comparison of subjects with right and left hemisphere lesions on measures of function, stroke severity, and lesion volume

	LHL		RHL		Test	*P*-Value
	n	Mean (SD)	n	Mean (SD)		
**Barthel**	12	16 (5)	16	17(4)	Rank Sum	1.0
**BIT**	12	136 (13)	18	128 (28)	Rank Sum	0.45
**NIHSS**	12	7 (4)	16	7 (5)	Rank Sum	0.69
**Lesion Volume**	5	38 (30)	13	60 (78)	Rank Sum	0.84

**Table 4.** Comparison of subjects with and without neglect on measures of function, stroke severity, and lesion volume

	No Neglect		Neglect		Test	P-Value
	n	Mean (SD)	n	Mean (SD)		
**Barthel**	13	18 (1)	3	12 (4)	Rank Sum	0.031*
**BIT**	13	141 (4)	5	95 (40)	t-test	0.062
**NIHSS**	12	5 (3)	4	13 (6)	t-test	0.002*
**Lesion Volume**	10	43 (72)	3	117 (80)	Rank Sum	0.05*

**Table 5.** Odds ratio predicting bilateral impairment

Subjects	Odds Ratio with 95% Confidence Interval
**All**	4.69 (1.99, 11.05)*
**Normal Controls**	7.94 (2.39, 11.05)*
**LHL & RHL**	1.28 (0.32, 5.13)
**LHL**	0.40 (0.04, 3.96)
**RHL**	2.67 (0.43, 16.39)

**Table 6.** Lesion volume is correlated with functional status and stroke severity

	Barthel	BIT	NIHSS
**Lesion Volume**	−0.45	−0.40	0.50
***p*-value**	0.06	0.10	0.04
**n-size**	17	18	17

**Table 7.** Stroke severity is correlated with percent failed sensory thresholds

	Barthel	BIT	NIHSS	Lesion Volume
**Overall % Fails**	−0.20	0.22	0.34 (p<0.07	−0.07
**ABT % Fails**	−0.18	0.03	0.39 (p<0.03)	0.20
**JND % Fails**	−0.10	0.28	0.12	−0.21
**ABT median Z**	−0.42 (*p*<0.08)			
