# Supplementary material for: On How Psychophysical Thresholds are Altered by Unilateral Brain Injury Due to Stroke
Source: Adv Neurol Neurosci Res. Author manuscript; Available in PMC 2022 Jan 11. (PMC8752079)
Supplement: FNNR-2-100014 Supplementary file [file NIHMS1757659-supplement-FNNR-2-100014_Supplementary_file.pdf]

Advances in Neurology and Neuroscience Research
Volume 2
Article ID: 100014
DOI: 10.51956/ANNR.100014

Research Article

On How Psychophysical Thresholds are Altered by Unilateral Brain Injury Due to Stroke

Melissa Allen[1], Tracy Kretzmer[2], George Jewell[3], Heather Murphy[4], Jeff Thostenson[5] and Mark Mennemeier[6]

[1]Department of Physical Therapy, University of Central Arkansas, USA

[2]Haley Veterans Hospital, USA

[3]Department of Neurology and Rehabilitation Medicine, University of Cincinnati, USA

[4]Clinical Solutions Group (CSG, Inc.), USA

[5]Department of Biostatistics, University of Arkansas for Medical Sciences, USA

[6]Department of Neurobiology and Developmental Sciences, University of Arkansas for Medical Sciences, USA

***Corresponding author**: Mark S. Mennemeier, Department of Neurobiology & Developmental Sciences, University of Arkansas for Medical Sciences, Little Rock, AR 72205, USA; E-mail: msmennemeier@uams.edu

Received: June 05, 2021; **Accepted**: July 24, 2021; **Published**: July 31, 2021

Copyright: ©2021 Allen M. This is an open access article distributed under the Creative Commons Attribution License, which permits unrestricted use, distribution, and reproduction in any medium, provided the original work is properly cited.

Supplementary Figures

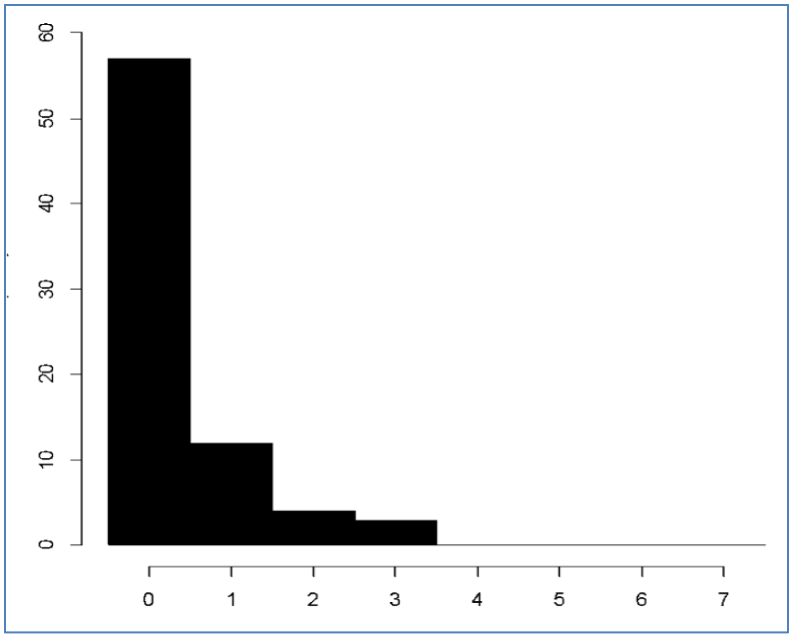

Supplemental Figure 1: Failed AB Thresholds – Normal Control Subjects

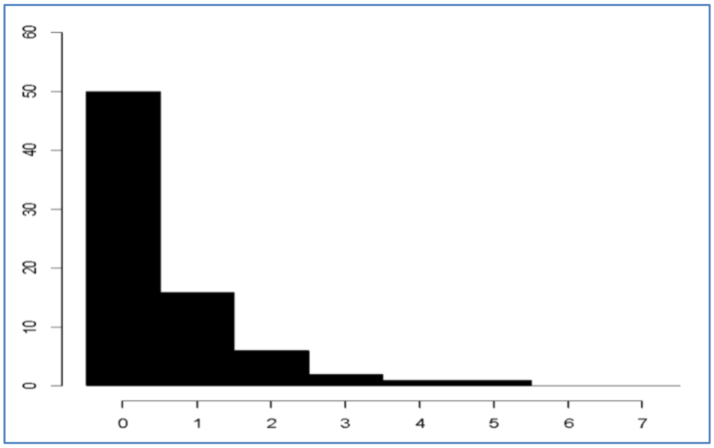

Supplemental Figure 2: Failed JND Thresholds - Normal Control Subjects

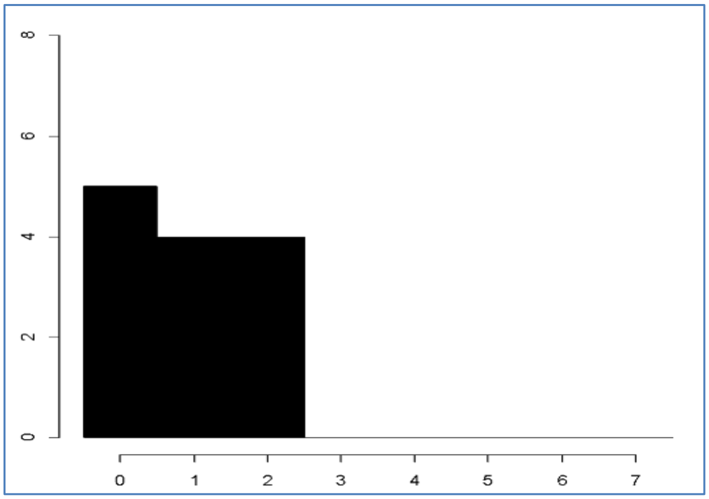

Supplemental Figure 3: Failed AB Thresholds - LHL Subjects

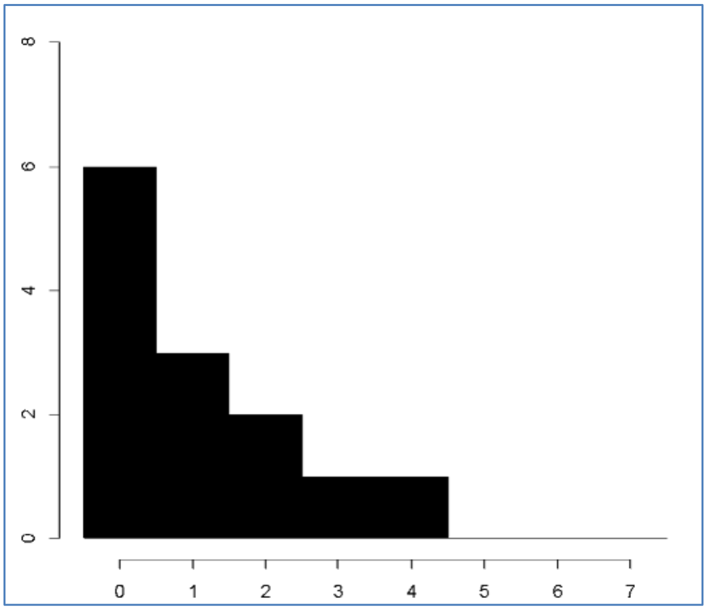

Supplemental Figure 4: Failed JND Thresholds - LHL Subjects

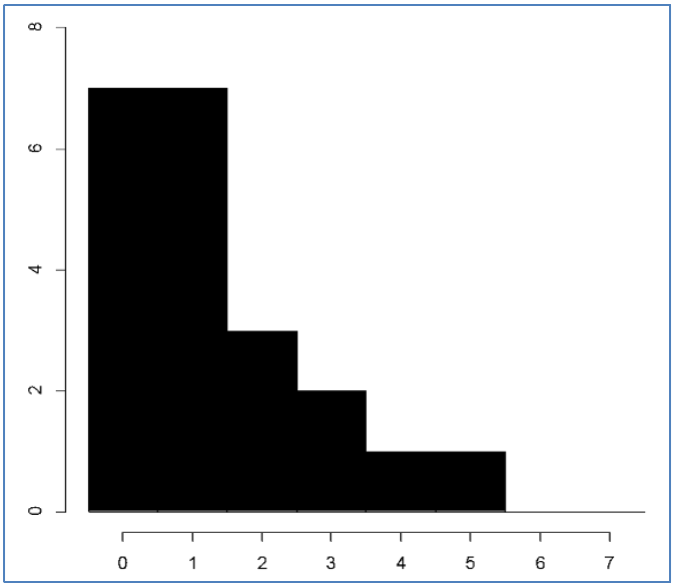

Supplemental Figure 5: Failed AB Thresholds - RHL Subjects

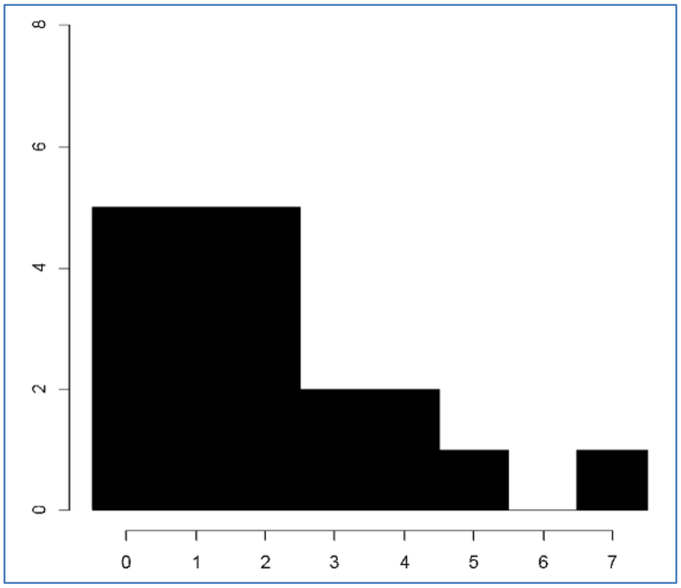

Supplemental Figure 6: Failed JND Thresholds - RHL Subjects
